# Leadless Pacemakers in Pediatric Patients: Is Less Actually More?

**DOI:** 10.19102/icrm.2020.111003

**Published:** 2020-10-15

**Authors:** Johannes C. von Alvensleben, Kathryn K. Collins

**Affiliations:** ^1^Children’s Hospital Colorado, University of Colorado School of Medicine, Aurora, CO, USA

**Keywords:** Leadless pacing, Micra, Nanostim, pediatric

Pediatric pacemaker implants currently make up less than 1% of all implants yet attempt to address the heterogeneous diagnoses found in adult patients while facing the additional technical concerns of small size and longer system requirements due to the young age of the recipients. The desire to avoid lead-related complications associated with transvenous systems influences the decision-making process. Infants and small children typically undergo epicardial pacemaker implantation to allow for growth and eventual transition to a transvenous system, although infant transvenous pacemakers are implanted at some centers.

The “holy grail” for pediatric patients remains a reliable leadless pacemaker and, with the introduction of the Micra™ (Medtronic, Minneapolis, MN, USA) and Nanostim™ (Abbott Medical, Chicago, IL, USA) systems, the potential to realize this broadly arrived. Leadless pacemakers are a self-contained generator and electrode system implanted directly into the right ventricle, typically via a femoral vein transcatheter approach. At the current time, they are restricted to ventricular pacing, although the most recent Micra™ version has VDD capabilities.

The performance of leadless-pacemaker implantation is largely limited to in adult patients, with only a limited number of case series and case reports involving pediatric patients published to date.^1–5^ In this issue of *The Journal of Innovations in Cardiac Rhythm Management*, Mahendran et al. present the successful implant and follow-up details of a Micra™ leadless pacemaker procedure conducted in a four-year-old male child weighing 16 kg. The authors describe using a traditional femoral venous approach with careful fluoroscopic and ultrasound assessment, in addition to serial dilation of the venous access point. They should be commended for their work as they describe their thoughts regarding patient selection, troubleshooting, and follow-up for what may be the smallest patient to date implanted with a leadless pacemaker.

Their work, however, also highlights several significant concerns of leadless pacemakers that are particularly applicable in pediatric patients and which temper the early enthusiasm. First is the large size of the introducer sheath (27-French outer diameter for the Micra™, 19.5-French for the Nanostim™), which is associated with complications during femoral access. In the reported case, a near-occlusive thrombus within the common femoral vein was noted despite procedural anticoagulation and, notably, vascular complications have been reported to occur in 0.7% of adult patients undergoing leadless pacemaker implant.^6^ Reports of adult and pediatric patients alike have described the use of jugular venous access to reduce vascular damage and improve device manipulation.^5^

More limiting is that only ventricular pacing is currently available with leadless systems, which severely restricts the indications available for pediatric patients. In their current state, leadless pacemakers appear most suitable for those who infrequently require ventricular pacing, have permanent atrial fibrillation with bradycardia, or have tachycardia–bradycardia syndrome. The recent addition of a VDD algorithm to the Micra™ platform was encouraging but its utility at heart rates above 115 bpm is questionable.^7^ There are no currently available options for leadless pacing in the atrium.

Battery longevity was initially reported to be approximately five to 15 years for both systems but the Nanostim™ has not yet gained approval from the Food and Drug Administration secondary to concerns of early battery depletion. Although a leadless pacemaker is theoretically retrievable, there is only limited experience reported with retrieval after chronic implantation and these devices can become encapsulated in cardiac tissue.^8,9^ The need for several potential lead extractions over the lifetime of a transvenous system implanted in childhood is frequently cited as a major limitation to eager adoption and, in their current state, leadless pacemakers do not effectively solve this problem. The fluoroscopic images presented by Mahendran et al. dramatically reveal the large size of the Micra™ system in relation to the patient’s right ventricular cavity. It is difficult to imagine that another device could be implanted if extraction proves unsuccessful.

In summary, leadless pacemakers present a tantalizing possibility to avoid the very real complications of traditional transvenous pacing systems, but significant concerns remain. Vascular complications secondary to the large delivery system are a possibility, albeit potentially obviated by a jugular approach in the smallest patients. Moreover, the absence of atrial pacing—or, at the very least, a viable VDD algorithm—limits the overall utility of these devices in pediatric patients at this time. Finally, concerns over battery longevity and the need for serial device extractions at a higher rate than that expected for transvenous leads necessitate the conduct of additional long-term investigations with the specific inclusion of pediatric patients.
